# Research Progress and Clinical Translation Potential of Coronary Atherosclerosis Diagnostic Markers from a Genomic Perspective

**DOI:** 10.3390/genes16010098

**Published:** 2025-01-18

**Authors:** Hanxiang Liu, Yuchen Zhang, Yueyan Zhao, Yuzhen Li, Xiaofeng Zhang, Lingyu Bao, Rongkai Yan, Yixin Yang, Huixian Zhou, Jinming Zhang, Siyuan Song

**Affiliations:** 1 School of Medical Imaging, Xuzhou Medical University, No. 209 Tongshan Road, Xuzhou 221004, China; 2 Medical and Information College, Xuzhou Medical University, No. 209 Tongshan Road, Xuzhou 221004, China; 3 Department of Surgery, University of Pittsburgh, Pittsburgh, PA 15260, USA; 4 Greenwich Hospital, Yale New Haven Health, Greenwich, CT 06519, USA; 5 Department of Internal Medicine, Montefiore Medical Center Wakefield Campus, 600 East 233rd Street, Bronx, NY 10466, USAhzhou@montefiore.org (H.Z.); 6 Department of Radiology, Ohio State University, Columbus, OH 43210, USA; 7 Department of Clinical Medicine, The First Clinical Medical College, Norman Bethune University of Medical Sciences, Jilin 130021, China; 8 Department of Integrative Biology and Pharmacology, McGovern Medical School, University of Texas Health Science Center at Houston (UTHealth), Houston, TX 77030, USA; 9 Department of Neuroscience, Baylor College of Medicine, Houston, TX 77030, USA

**Keywords:** coronary atherosclerosis, genomics, gene markers, genome-wide association study, transcriptomics, epigenomics, precision medicine, early diagnosis

## Abstract

**Objective:** Coronary atherosclerosis (CAD) is characterized by arterial intima lipid deposition, chronic inflammation, and fibrous tissue proliferation, leading to arterial wall thickening and lumen narrowing. As the primary cause of coronary heart disease and acute coronary syndrome, CAD significantly impacts global health. Recent genetic studies have demonstrated CAD’s polygenic and multifactorial nature, providing molecular insights for early diagnosis and risk assessment. This review analyzes recent advances in CAD-related genetic markers and evaluates their diagnostic potential, focusing on their applications in diagnosis and risk stratification within precision medicine. **Methods:** We conducted a systematic review of CAD genomic studies from PubMed and Web of Science databases, analyzing findings from genome-wide association studies (GWASs), gene sequencing, transcriptomics, and epigenomics research. **Results:** GWASs and sequencing studies have identified key genetic variations associated with CAD, including *JCAD/KIAA1462*, *GUCY1A3*, *PCSK9*, and *SORT1*, which regulate inflammation, lipid metabolism, and vascular function. Transcriptomic and epigenomic analyses have revealed disease-specific gene expression patterns, DNA methylation signatures, and regulatory non-coding RNAs (miRNAs and lncRNAs), providing new approaches for early detection. **Conclusions:** While genetic marker research in CAD has advanced significantly, clinical implementation faces challenges including marker dynamics, a lack of standardization, and integration with conventional diagnostics. Future research should prioritize developing standardized guidelines, conducting large-scale prospective studies, and enhancing multi-omics data integration to advance genomic diagnostics in CAD, ultimately improving patient outcomes through precision medicine.

## 1. Introduction

Coronary atherosclerosis (CAD) is the primary pathological basis for coronary heart disease (CHD) and acute coronary syndrome (ACS), and it is one of the leading global causes of death and disease burden. Statistics show that CAD causes nearly 10 million deaths worldwide annually, accounting for the largest proportion of cardiovascular-related mortality. This chronic arterial disease is characterized by lipid deposition, chronic inflammation, and fibrous tissue proliferation, which progressively lead to arterial sclerosis, lumen narrowing, and even complete occlusion [1]. Although traditional risk assessment models and diagnostic tools (such as the Framingham Risk Score, electrocardiography, and coronary angiography) play critical roles in the diagnosis and treatment of mid-to-late-stage CAD, they are less effective in identifying the early, asymptomatic stages of the disease. With the advent of precision medicine, individualized molecular-level diagnostics have become a research focus, offering new hope for the early detection and risk stratification of CAD [2,3].

In recent years, the rapid development of genomics has provided novel perspectives and tools for CAD research. By studying the structure, function, and disease associations of genetic material, genomics has offered important insights into the pathogenesis and potential molecular pathways of CAD [4]. For instance, genome-wide association studies (GWASs) have become a core technology in CAD genetics research. By scanning genetic variations across large populations, GWASs have identified several key genetic loci related to CAD, such as *JCAD/KIAA1462*, *GUCY1A3*, and *PCSK9* [5,6]. These genes are closely associated with vascular endothelial function regulation, lipid metabolism, and inflammatory responses, laying the foundation for developing diagnostic and therapeutic targets [7,8]. Furthermore, gene sequencing technologies (including second- and third-generation sequencing) have revealed critical mutations and genetic variations associated with CAD [9]. Notably, these discoveries have already yielded clinical applications, with *PCSK9* inhibitors now serving as effective therapeutic agents for lowering cholesterol in high-risk CAD patients [10].

Transcriptomics and epigenomics, as extended fields of genomics, have deepened our understanding of the molecular mechanisms underlying CAD. Transcriptomic studies have discovered significantly abnormal mRNA expression levels of certain key genes in CAD-affected tissues, such as those involved in inflammation and oxidative stress. Additionally, epigenomics, through the study of DNA methylation, histone modification, and non-coding RNAs (e.g., miRNAs and lncRNAs), has highlighted the critical role of epigenetic changes in CAD pathogenesis [11]. For example, research shows that the DNA methylation status of specific gene regions is significantly altered in CAD patients, and the expression patterns of certain miRNAs are strongly correlated with disease severity, providing a basis for developing potential biomarkers [12].

Despite these advances in genomic research, several challenges impede clinical implementation. For example, CAD is a dynamically evolving process involving different genes and molecular mechanisms at various stages of the disease [13]. The expression levels and functions of genetic markers may change throughout disease progression, limiting the universal applicability of single markers in clinical diagnostics. Furthermore, the lack of standardized diagnostic criteria and guidelines poses a significant barrier, as different studies and medical institutions employ inconsistent methods and thresholds for genetic testing, hindering the broad application of genomics findings [14]. Additionally, integrating genomics with traditional diagnostic methods, such as imaging and biochemical markers, to develop multidimensional diagnostic strategies requires further exploration [15].

Against this backdrop, this review aims to systematically summarize the research progress of CAD-related genetic markers, exploring their applications in early diagnosis, risk stratification, and personalized treatment from multiple perspectives, including GWASs, gene sequencing, transcriptomics, and epigenomics. It also analyzes the challenges related to dynamic marker changes, standardized diagnostic methods, and technical integration, proposing future directions and strategies to promote the clinical translation of genomic diagnostics in CAD. Ultimately, these efforts aim to achieve precision medical management of the disease and improve patient outcomes [16].

## 2. Research Methodology

### 2.1. Literature Search Strategy

Authoritative databases such as PubMed, Web of Science, Wiley Online Library, and ScienceDirect were used to conduct the search until June 2024 to obtain the research progress and cutting-edge results in the field of coronary atherosclerosis genomics. Disease terms such as “coronary atherosclerosis”, “coronary heart disease”, “atherosclerosis”, “genomics”, “genetic markers”, and “genomics” were used. Using “gene marker” and other genomics terms and “*JCAD/KIAA1462*” and other key gene names, we constructed a multi-group search formula through logical operators, such as “(coronary atherosclerosis OR coronary heart disease OR atherosclerosis) AND (genomics OR gene markers OR genome-wide association study OR gene sequencing OR transcriptomics OR epigenomics) AND (*JCAD/KIAA1462* OR *GUCY1A3* OR *PCSK9* OR *SORT1*)”, to conduct a comprehensive search.

### 2.2. Literature Screening Criteria

Studies were included if they directly investigated the genomic aspects of coronary atherosclerosis, including genetic markers for diagnosis and risk stratification, molecular pathogenesis, and applications of genomic technologies. Each study was required to provide a clear methodological description, well-documented results, and valid conclusions supported by data.

Studies were excluded if they examined cardiovascular diseases without a genomic analysis of coronary atherosclerosis or primarily focused on non-genomic factors (environmental, lifestyle) without a genetic mechanism investigation. Studies with an inadequate sample size, lack of appropriate controls, or unsound methodology were also excluded. For multiple publications using the same dataset, only the most recent or comprehensive study was included.

## 3. Pathogenesis of Coronary Atherosclerosis

**Figure 1:**
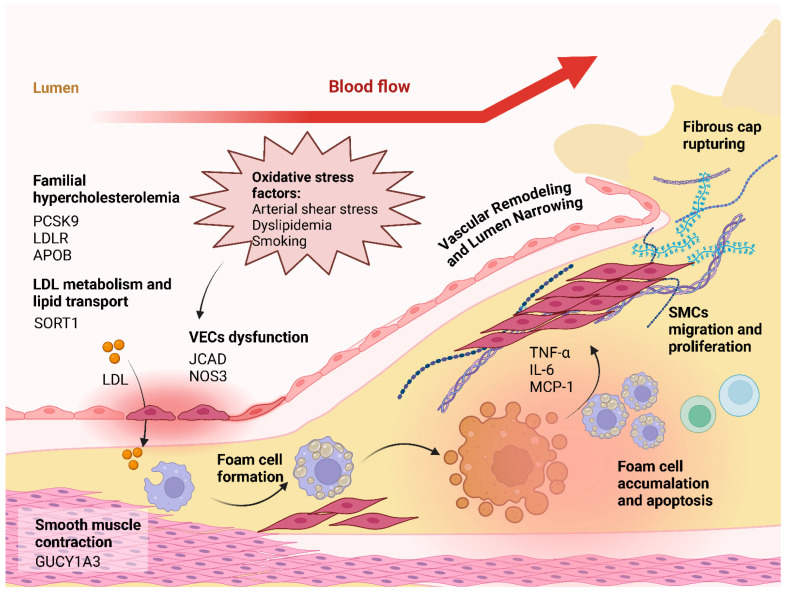
Pathological cascade in coronary atherosclerosis development. This figure illustrates the progression of coronary atherosclerosis, beginning with vascular endothelial cell (VEC) dysfunction caused by genetic predispositions (e.g., *JCAD*, *NOS3*), oxidative stress factors (arterial shear stress, dyslipidemia, and smoking), and familial hypercholesterolemia (*PCSK9*, *LDLR*, *APOB*). LDL infiltration into the arterial wall promotes foam cell formation, driven by impaired LDL metabolism (*SORT1*). The resulting inflammatory response involves cytokines such as TNF-α, IL-6, and MCP-1, which stimulate smooth muscle cell (SMC) proliferation and migration. These processes contribute to vascular remodeling, lumen narrowing, and fibrous cap formation, which may ultimately lead to rupture, foam cell apoptosis, and advanced atherosclerotic lesions.

The pathogenesis of coronary atherosclerosis, a complex and serious threat to human cardiovascular health, involves the interaction and dynamic evolution of multiple factors (Figure 1).

### 3.1. Early Vascular Functional Alterations and Inflammation Initiation

Risk factors including genetic predisposition (*JCAD*, *NOS3*), oxidative stress, and familial hypercholesterolemia (*PCSK9*, *LDLR*, *APOB*) initiate vascular endothelial cell (VEC) dysfunction. Additional contributors include age, lifestyle factors, chronic diseases, infections, and autoimmune conditions [17].

The inflammatory cascade begins when compromised VEC barrier function allows LDL penetration into the arterial intima. Oxidized LDL triggers the endothelial expression of adhesion molecules (ICAM-1, VCAM-1), promoting monocyte adhesion and migration. Released inflammatory mediators (MCP-1, IL-6, TNF-α) drive monocyte-to-macrophage differentiation and smooth muscle cell (SMC) phenotype transformation [17,18,19].

### 3.2. Vascular Remodeling and Lesion Progression

Upon activation of the inflammatory response, SMCs migrate and proliferate from the intima to the endothelium as an adaptive response to injury aimed at repairing the damaged vessel wall. SMCs migrating to the intima synthesize and secrete collagen, elastic fibers, and other extracellular matrix (ECM) components, which cause the vessel wall to thicken and the lumen to narrow. At the same time, ECM remodeling occurs, and the balance of matrix metalloproteinases (MMPs) and their inhibitors (TIMPs) is disrupted, with a relative increase in the activity of MMPs, which degrade ECM components and change the structure and composition of the vessel wall, an important feature of vascular remodeling [20].

Macrophages phagocytose ox-LDL to form foam cells, which accumulate in large numbers under the intima and release inflammatory mediators, forming an inflammatory microenvironment. This exacerbated the inflammatory response, further promoted the proliferation and migration of SMCs, and accelerated vessel wall thickening and lumen narrowing. Foam cell apoptosis releases lipids and cellular debris to form lipid cores, which increase plaque size and worsen the structural and functional damage of the vessel wall [21].

### 3.3. Persistent Effects of Plaque Formation and Vascular Remodeling

With lesion progression, the ECM secreted by SMCs encapsulates lipid cores to form an atheromatous plaque fibrous cap. Fibrous cap thickness and stability are critical for plaque development. In this process, vascular remodeling continues. On the one hand, SMCs continue to proliferate and synthesize the ECM to thicken the fibrous cap in order to stabilize the plaque; on the other hand, the continuous action of MMPs may degrade the fibrous cap to make it thinner, and the stability of the plaque decreases.

The structure and function of the vessel wall are constantly changing in vascular remodeling. Intimal thickening, luminal narrowing, and plaque formation affect local hemodynamics, resulting in changes in blood flow velocity and shear stress. Slowed blood flow and low-shear-stress regions promote lipid deposition and inflammatory cell aggregation, exacerbating vessel wall injury; high-shear-stress regions promote the proliferation of SMCs and thickening of the fibrous cap, which maintains the integrity of the vessel wall, creating a vicious cycle [22].

### 3.4. Plaque Progression and Clinical Consequences

Over time, unstable plaques form with thin fibrous caps, lipid-rich cores, and significant inflammatory cell infiltration. The unstable plaque is prone to rupture in response to triggers such as hemodynamic fluctuations and increased inflammatory response. After rupture, the lipid core and tissue factor activate platelet aggregation and the coagulation system to form thrombus, leading to acute lumen occlusion and triggering acute coronary syndromes (ACSs), such as myocardial infarction and other serious cardiovascular events [23].

Although stable plaques are relatively stable, the increase in volume will also aggravate coronary artery stenosis, leading to insufficient blood supply to the myocardium, causing chronic myocardial ischemia symptoms, such as stable angina pectoris, affecting the quality of life and prognosis of patients.

In summary, vascular remodeling occurs early in the pathogenesis of coronary atherosclerosis, and it is intertwined and synergistic with lipid deposition and inflammatory responses to drive disease progression and ultimately lead to cardiovascular complications. An in-depth understanding of this process is important for the early identification and intervention of atherosclerosis to reduce the risk of cardiovascular disease.

## 4. Role of Genetic Factors in Coronary Atherosclerosis

### 4.1. Core Role of Genetic Factors

**Figure 2:**
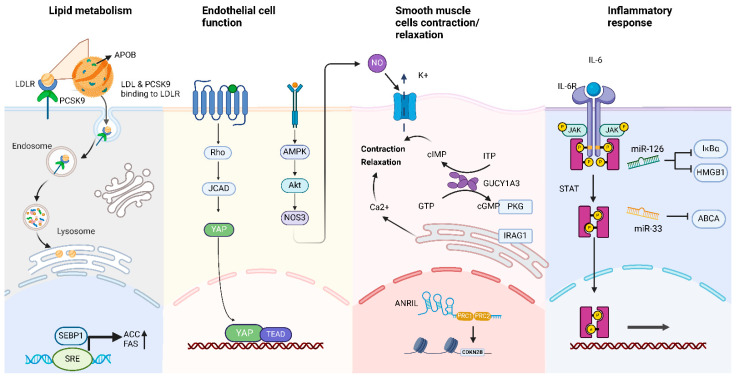
GWAS-identified genes and their role in coronary artery disease pathophysiology. This figure highlights the molecular pathways involving key genes identified through GWASs that contribute to coronary artery disease (CAD). Lipid metabolism: Genes such as *PCSK9* and *LDLR* regulate low-density lipoprotein (LDL) cholesterol levels. Gain-of-function mutations in *PCSK9* lead to hypercholesterolemia and increased CAD risk by affecting LDL uptake and degradation. Endothelial cell function: The *JCAD/KIAA1462* gene encodes a protein critical for endothelial cell adhesion and vascular integrity. Variants impair endothelial cell function, promoting CAD development. Smooth muscle contraction/relaxation: The *GUCY1A3* gene encodes soluble guanylyl cyclase (sGC), a mediator of smooth muscle relaxation. Mutations disrupt vascular tone and contribute to CAD risk. Inflammatory response: Genes like *IL6R* influence cytokine signaling, contributing to vascular inflammation and lesion progression. MicroRNAs such as *miR-126* and *miR-33* further modulate inflammatory and lipid transport pathways.

Genetic factors play a crucial role in the development and progression of coronary atherosclerosis. Studies of twins and familial cases have shown that genetic factors account for approximately 40–60% of the risk of developing CAD [24]. GWASs have further identified hundreds of genetic variants associated with CAD, highlighting the significant role of genetic susceptibility in individual differences in disease risk [25] (Figure 2).

### 4.2. Key Genes and Their Functions

Several key genes play important roles in the pathogenesis of coronary atherosclerosis. Among them, the *JCAD/KIAA1462* gene encodes a protein closely related to endothelial cell adhesion [26]. Mechanistic studies have elucidated *JCAD*’*s* molecular pathway in endothelial cells. Research has revealed that *JCAD* forms a crucial interaction with *LATS2* (large tumor suppressor kinase 2), which serves as a key regulatory component of the Hippo signaling pathway. Through this interaction, *JCAD* functions as a negative regulator of Hippo signaling [27]. This negative regulation results in enhanced activity of *YAP* (yes-associated protein), which acts as the primary transcriptional effector of the pathway [28].

*GUCY1A3* encodes the α1-subunit of soluble guanylyl cyclase (sGC), which produces cyclic guanosine monophosphate (cGMP), triggering multiple cellular responses including platelet aggregation inhibition and smooth muscle cell relaxation, serving as a central enzyme in cardiovascular regulation [29]. A genome-wide association study meta-analysis identified *NOS3*, which encodes endothelial NO synthase as a CAD risk gene [30]. Furthermore, genetic studies have demonstrated the NO-sGC-cGMP pathway’s role in mediating both CAD and myocardial infarction risk. Notably, genetic variants at the *GUCY1A3* locus have been shown to influence *NOS3* expression [29].

*PCSK9*’*s* influence on CAD operates through multiple mechanisms, including binding to epidermal growth factor (EGF) domains on receptors, interacting with receptors in lipid rafts and cell membrane micro-domains, and modulating gene expression and protein responses involved in cardiac complications [31]. Most importantly, the protein’s interaction with *LDLR* follows two distinct pathways, namely extracellularly, where circulating *PCSK9* binds to *LDLR*’*s* EGF-A domain, forming a complex that undergoes endocytosis and lysosomal degradation, preventing *LDLR* recycling and increasing plasma LDL-c levels; and intracellularly, where *PCSK9* binds *LDLR* in the trans-Golgi network, directing it to lysosomes before reaching the cell surface—a process requiring *PCSK9*’*s* catalytic activity. Mutations in this gene have a significant effect on LDL-c metabolism [32]. Except for the role in lipid metabolism, *PCSK9*, also associated with expression of apoptosis-related proteins such as Bax and caspase-3, possibly induces the apoptosis of endothelial cells and stimulates the differentiation of vascular smooth muscle cells into a synthetic phenotype [33].

### 4.3. Genetic Association with Familial Hypercholesterolemia

Familial hypercholesterolemia (FH) is a monogenic hereditary disorder typically caused by loss- or gain-of-function mutations in the *LDLR*, *APOB*, or *PCSK9* genes. FH patients exhibit significantly elevated plasma LDL levels, making them a high-risk group for early-onset coronary atherosclerosis. These findings reveal the direct contribution of monogenic mutations to CAD risk [34].

### 4.4. Polygenic Cumulative Effects

CAD is not determined by a single gene but driven by the cumulative effects of multiple genes and environmental factors. Many risk loci identified through GWASs contribute modestly individually, but their combined effects can significantly increase disease risk [35]. Studies using polygenic risk scores (PRSs) have shown that individuals with high genetic risk have a significantly higher incidence of CAD, even under similar environmental conditions [36].

### 4.5. Epigenetic Regulation

Genetic susceptibility is not only reflected in gene mutations and variations but also involves epigenetic regulatory mechanisms. For example, abnormal changes in DNA methylation levels have been identified as an important regulatory mechanism in CAD [37]. Additionally, the dysregulated expression of certain miRNAs (e.g., mi*R-126* and *miR-33*) may contribute to CAD pathogenesis by modulating lipid metabolism and inflammatory responses [38].

## 5. Applications of Genomic Research Methods in the Diagnosis of Coronary Atherosclerosis

### 5.1. Genome-Wide Association Studies (GWASs)

#### 5.1.1. Evolution and Rationale of GWASs in CAD Research

Early GWASs of coronary atherosclerosis had significant limitations. Small, geographically restricted sample sizes compromised genetic diversity and generalizability, while first-generation genotyping platforms had poor SNP detection capabilities. Despite strict statistical thresholds (*p* < 5 × 10^−8^), results were often unreliable and difficult to validate independently [39].

Recent GWASs in CAD have made significant progress, largely driven by the analysis of expanded cohorts [40]. A comprehensive genome-wide association analysis of 3221 cardiovascular patients led to the identification of novel candidate SNPs in *BAAT*, *BCL3*, and *CMTM6* genes. These genetic variants were found to have direct functional impacts on cholesterol levels and LDL biosynthesis, particularly in relation to patients’ responses to statin therapy in CAD treatment, highlighting the clinical relevance of these genetic discoveries [41].

New challenges have emerged regarding genetic structure differences because of population stratification. These differences can generate false positive or false negative results that affect the accuracy of research conclusions [39]. Several methods that correct for stratification have been studied, including detecting stratification, inferring genetic ancestry, family-based association tests, mixed models, and low-frequency and rare variants. Current studies must develop robust methodologies to properly address this statistical challenge and ensure reliable findings [42].

#### 5.1.2. Key CAD Genes Identified by GWASs and the Value of Their Detection

Genome-wide association studies (GWASs) have identified several genes strongly associated with coronary artery disease (CAD). Four genes in particular—*JCAD/KIAA1462*, *GUCY1A3*, *PCSK9*, and *SORT1*—have emerged as especially significant. Based on a comprehensive literature review, we analyzed these genes’ characteristics and evaluated their potential clinical applications.

*JCAD/KIAA1462*: *JCAD*, previously known as *KIAA1462*, represents a promising therapeutic target for cardiovascular disease intervention [43]. Multiple genome-wide association studies have consistently identified *JCAD* as a significant risk locus for coronary artery disease [26,44]. The STARNET study (Stockholm-Tartu Atherosclerosis Reverse Networks Engineering Task) revealed a key CAD-associated single nucleotide polymorphism, *rs2487928*, which functions as a highly significant expression quantitative trait locus (eQTL) for *JCAD* in the arterial wall and atherosclerotic aortic root [45]. Research indicates this variant may interfere with normal endothelial cell function and disrupt physiological processes, thereby elevating coronary heart disease risk [28,44,46].

Research by Hara and colleagues has demonstrated that *JCAD* plays a crucial role in vascular function. Their studies showed that *JCAD* knockdown reduces angiogenic processes and decreases the vascular endothelial growth factor-induced phosphorylation of both extracellular signal-regulated kinase and VEGF receptor 2 (VEGFR2). These findings suggest that *JCAD*’*s* influence extends beyond coronary artery disease, potentially affecting tumor growth through its impact on vascular development [47].

*GUCY1A3:* As previously described, *GUCY1A3* plays a crucial role in CAD vasoregulatory mechanisms and can be analyzed through PCR and the sequencing of specific gene fragments in peripheral blood. Research by Kessler et al. revealed that the intronic variant *rs7692387* contains a DNase I site, where ZEB1 transcription factor preferentially binds to the non-risk allele, leading to increased *GUCY1A3* expression and sGC activity, with higher sGC expression correlating to reduced atherosclerosis risk in both humans and mice. Their study also found that carriers of the homozygous *GUCY1A3* risk allele have an elevated risk of cardiovascular death or stent thrombosis within 30 days after coronary stenting, possibly due to increase on-aspirin platelet reactivity [29]. Kessler et al. also demonstrated that carriers of homozygous *GUCY1A3* risk allele face higher risks of cardiovascular death or stent thrombosis within 30 days after coronary stenting, potentially due to an increase in on-aspirin platelet reactivity [48]. Further investigation by Hall et al., through two randomized placebo-controlled trials in primary prevention, demonstrated that aspirin reduced CVD events in individuals homozygous for the *GUCY1A3* risk allele, while heterozygote individuals experienced more events when taking aspirin [49]. These findings collectively underscore *GUCY1A3*’*s* significance in pathological CAD progression.

*PCSK9:* Lipid metabolism dysfunction plays a central role in CAD progression, with *PCSK9* emerging as a key clinical target for treating hypercholesterolemia due to its deep involvement in lipid metabolism.

Several studies were conducted to verify the efficacy and safety of the *PCSK9* inhibitors in treating dyslipidemia and cardiovascular disease. The development of *PCSK9* inhibitors has progressed through three generations, namely first-generation monoclonal antibodies (evolocumab and alirocumab) with proven efficacy and safety in follow-up studies [50,51], second-generation small-interfering RNA (inclisiran) validated through phase III trials and ongoing cardiovascular outcome studies [52], and third-generation approaches including oral drugs (MK-0616), fusion protein (lerodalcibep), vaccination, and gene-editing strategies [53].

Beyond lipid regulation, *PCSK9* influences inflammatory pathways crucial to atherosclerosis development. It regulates TLR4 activation through MyD88 and TRIF pathways (predominantly MyD88), inducing *PCSK9* transcription in the aorta and activating NF-kB, which affects apoptosis and autophagy [54]. Additionally, research by Ding et al. revealed that recombinant *PCSK9* enhances the expression of multiple scavenger receptors (SRs), particularly *LOX-1*, *SRA*, and *CD36*, with *LOX-1* showing the highest upregulation [55]. These SRs facilitate ox-LDL uptake by macrophages during early atherosclerosis, demonstrating *PCSK9*’*s* intertwined role in inflammation and its significance in atherosclerosis progression and subsequent myocardial ischemia.

*SORT1: SORT1* plays a crucial role in lipid metabolism through its regulation of hepatic VLDL secretion and plasma LDL-C levels. Research by Strong et al. demonstrated that hepatic sortilin overexpression reduces plasma cholesterol by decreasing VLDL secretion, while knockout models show increased VLDL secretion [56]. The gene’s importance is highlighted by genome-wide association studies identifying the 1p13 locus containing *SORT1*, as strongly associated with both LDL-C levels and myocardial infarction risk [57]. SNPs in this region, particularly *rs12740374*, create a C/EBP transcription factor binding site that alters *SORT1* expression. Additionally, *SORT1* functions in post-translational *PCSK9* regulation by facilitating its secretion, forming an important regulatory network in lipid metabolism. Recent therapeutic developments include antisense oligonucleotides targeting *SORT1* for potential cardiovascular disease treatment, though these approaches are still in experimental stages [58]. These findings collectively emphasize *SORT1*’*s* significance in both lipid regulation and cardiovascular disease pathogenesis.

#### 5.1.3. Progress and Challenges of GWASs in Advancing the Clinical Use of CAD

GWASs have fundamentally transformed our understanding of CAD genetics, revealing its complex polygenic nature. While individual genetic variants show modest effects, their cumulative impact significantly influences disease susceptibility and progression [4].

Recent advances in PRSs have demonstrated promising clinical applications. A landmark 10-year longitudinal study of 288 patients revealed that a high PRS correlated with increased baseline plaque volume and accelerated progression. Each standard deviation increase in the PRS led to a 0.69% greater plaque volume progression per decade after multivariate adjustment. The integration of PRSs with traditional risk factors improved predictive accuracy for non-calcified plaque progression (AUC: 0.73 vs. 0.69, *p* = 0.039) [59]. Meta-analyses across multiple cohorts have consistently shown that individuals with a high PRS have a higher risk of CAD compared to those with low scores [60].

Machine learning approaches have further enhanced PRS accuracy. Advanced algorithms incorporating both genetic and clinical variables have achieved improved risk stratification, particularly in identifying high-risk individuals who might benefit from early intervention. These computational advances have enabled a more sophisticated modeling of gene–environment interactions and better account for population-specific genetic architectures.

However, significant challenges persist in translating GWAS findings to clinical practice. Technical barriers include platform heterogeneity in genotyping arrays, which creates systematic differences that imputation methods only partially address [39]. Statistical challenges remain in controlling false positive results and managing multiple testing burden, despite advances in PRS construction methodology. Clinical implementation faces additional hurdles, including the need for standardized testing protocols, provider education, and clear guidelines for risk communication [61].

Recent research has focused on developing more robust statistical frameworks and validation strategies. The integration of PRSs with established risk assessment tools has shown promise in improving risk stratification accuracy [62]. A population-specific calibration of PRSs and the consideration of ancestry-specific genetic architectures have emerged as crucial factors in improving predictive accuracy across diverse populations.

Looking forward, the field is moving toward more comprehensive risk assessment models that integrate multiple data types, including genetic, clinical, and environmental factors. Forrest et al. demonstrated a machine learning-based ISCAD model’s effectiveness in large biobanks (*n* = 95,935), achieving AUCs of 0.91–0.95. The model showed strong clinical correlation, with coronary stenosis increasing 12% per ISCAD quartile and mortality risk escalating across deciles (HR 1.0–56). Notably, 46% of undiagnosed high-ISCAD individuals showed clinical CAD evidence per ACC/AHA guidelines, highlighting its utility in subclinical detection [63]. Success in addressing these challenges could revolutionize CAD prevention and management, enabling truly personalized cardiovascular medicine.

### 5.2. Genetic Sequencing Analysis

#### 5.2.1. Overview of Genetic Sequencing Technologies and Their Characteristics

Common genetic sequencing technologies include first-generation sequencing (Sanger sequencing), second-generation sequencing (e.g., Illumina sequencing), and third-generation sequencing (e.g., PacBio sequencing). Each has unique features in sequencing throughput, read length, accuracy, and cost, suitable for different research scenarios [64]. (1) First-generation sequencing (Sanger): high accuracy but low throughput and relatively high cost. (2) Second-generation sequencing (Illumina): high throughput and low cost, ideal for large-scale studies. (3) Third-generation sequencing (PacBio): long read lengths and high resolution, particularly useful for structural variant (SV) detection. As sequencing technologies advance, second- and third-generation sequencing are increasingly used complementarily. The high throughput and low cost of second-generation sequencing combined with the long-read and high-resolution capabilities of third-generation sequencing enable a comprehensive analysis of CAD’s genetic basis.

#### 5.2.2. Applications of Genetic Sequencing in CAD Diagnosis

(1) Whole-Genome Sequencing (WGS) for Identifying Key Mutations

Large-scale WGS studies in CAD patients have identified non-coding region variants significantly associated with CAD. For example, SNPs in the enhancer region of the *SORT1* gene have been shown to alter lipid metabolism-related gene expression, affecting CAD risk [65].

(2) Whole-Exome Sequencing (WES) for Identifying Pathogenic Mutations

WES has been widely used to analyze rare variants associated with familial CAD. For example, studies on familial hypercholesterolemia patients identified a loss-of-function mutation in the *LDLR* gene, closely related to elevated LDL-C levels, providing a precise therapeutic target.

(3) Long-Read Sequencing for Structural Variant Detection

Third-generation sequencing excels in detecting structural variants (SVs) and copy number variations (CNVs). For example, PacBio sequencing identified a large deletion in the *GUCY1A3* gene region, closely linked to smooth muscle dysfunction and increased CAD risk.

(4) Liquid Biopsy for Precision Diagnosis

Second-generation sequencing has also been applied in liquid biopsies, detecting circulating cell-free DNA (cfDNA) mutations in blood for early CAD diagnosis. For instance, studies show that CAD patients exhibit elevated frequencies of *PCSK9* mutations in plasma cfDNA, which could serve as a potential biomarker for disease progression [66].

Genetic sequencing has become a core tool for CAD research and diagnosis. The integrated use of different sequencing technologies allows researchers to comprehensively analyze CAD’s genetic basis at genomic, transcriptomic, and structural levels, supporting early diagnosis, risk stratification, and individualized treatment. However, challenges such as sequencing costs, data complexity, and clinical translation need further exploration and optimization.

Transcriptomic techniques have also been extended to analyze plasma and exosomal RNA, providing non-invasive diagnostic tools for CAD. For example, one study found that inflammatory gene mRNA (e.g., *CCL2*) expression was significantly upregulated in plasma exosomes of CAD patients, suggesting its potential as a marker for early detection [66].

### 5.3. Applications and Discoveries of Transcriptomics in CAD Diagnosis

#### 5.3.1. Key Genes and Pathways Identified by Transcriptomics

(1) Inflammation-Related Genes

RNA-seq studies have shown a significant activation of inflammatory factors (e.g., IL6, TNF-α) and their related signaling pathways in the arterial wall tissues of CAD patients. For instance, elevated *IL6R* expression is closely associated with local inflammatory responses, serving as a key driver of CAD plaque formation and rupture [67].

(2) Lipid Metabolism Genes

Transcriptomics has revealed the abnormal expression of several lipid metabolism-related genes in CAD [68]. For example, the upregulation of *APOB* and *SREBF1* may exacerbate cholesterol accumulation and promote the progression of atherosclerotic lesions.

(3) Vascular Function Genes

RNA-seq analysis also identified significant abnormalities in genes related to vascular smooth muscle and endothelial function. The reduced expression of *NOS3*, for instance, may lead to decreased nitric oxide synthesis, further aggravating vascular constriction and dysfunction [69].

(4) Identification of Potential Biomarkers for Early Diagnosis

Transcriptomics data assist in identifying potential molecular markers for the early diagnosis of CAD, such as the following:

*CDKN2B-AS1 (ANRIL)*: A long non-coding RNA (lncRNA) associated with CAD. Its expression is significantly upregulated and may influence smooth muscle cell proliferation by regulating cyclin-dependent kinase inhibitors [70].

*MIR-33*: This miRNA, linked to lipid metabolism and inflammation, shows abnormal expression in CAD patients and may serve as a key molecular marker for disease diagnosis and therapeutic response evaluation [71].

#### 5.3.2. Transcriptomic Features of Disease Severity

RNA-seq studies have revealed significant transcriptional differences across different stages of CAD (e.g., early lipid plaques, stable plaques, and unstable plaques). For instance, the following have been observed:

(1) Unstable plaques: an elevated expression of matrix metalloproteinases (e.g., MMP9) suggests an increased risk of plaque rupture.

(2) Stable plaques: an increased expression of collagen-related genes (e.g., *COL1A1* and *COL3A1*) indicates enhanced mechanical strength of the plaques.

Transcriptomics provides a wealth of resources for understanding the molecular mechanisms of CAD and developing diagnostic biomarkers [72]. Using techniques like RNA-seq, researchers have not only uncovered gene expression changes associated with CAD progression but also identified several molecular markers with diagnostic and therapeutic potential. However, translating these findings into clinically applicable diagnostic tools and further exploring the specific transcriptional characteristics of different disease stages require deeper research and technological innovation.

### 5.4. Epigenomics

#### 5.4.1. Core Research Areas of Epigenomics

Epigenomics primarily studies mechanisms such as DNA methylation, histone modifications (e.g., methylation, acetylation), and non-coding RNAs (e.g., miRNAs, lncRNAs) that regulate gene expression [73]. These epigenetic modifications affect cellular physiological functions and the development of diseases by altering gene transcription activity and chromatin structure without changing the DNA sequence.

#### 5.4.2. Applications and Discoveries of Epigenomics in CAD Diagnosis

Epigenomics provides new insights into the early diagnosis and risk assessment of coronary atherosclerosis (CAD). Large-scale epigenomic studies have identified multiple epigenetic markers closely associated with CAD progression.

#### 5.4.3. Diagnostic Applications of DNA Methylation

(1) Specific gene methylation patterns: studies have shown that the DNA methylation levels of specific genes (e.g., *APOB*, *NOS3*) in the plasma or lesions of CAD patients are significantly upregulated or downregulated, making them potential biomarkers for disease diagnosis.

(2) Predicting disease progression: for instance, a study on CAD patients revealed that the methylation status of the *DNMT3B* gene is significantly associated with arterial plaque instability, providing molecular evidence for risk prediction [74].

#### 5.4.4. Diagnostic Potential of miRNAs

(1) Plasma miRNA detection: numerous studies have reported significantly elevated levels of inflammation-related miRNAs (e.g., mi*R-146a*, *miR-21*) in the plasma of CAD patients, indicating their potential as non-invasive biomarkers for early disease detection [75].

(2) Therapeutic response evaluation: changes in the expression levels of certain miRNAs are strongly associated with reduced inflammatory responses following drug interventions (e.g., statins), suggesting their application in monitoring treatment efficacy.

(3) Role of lncRNAs in risk stratification: the expression levels of long non-coding RNAs (lncRNAs), such as *ANRIL*, are significantly associated with plaque stability and cardiovascular event risk in CAD patients [76]. For example, *ANRIL* regulates the *CDKN2B* gene, influencing smooth muscle cell proliferation and inflammatory responses, making it a critical candidate marker for CAD risk stratification.

#### 5.4.5. Histone Modification Mechanisms and Biomarker Development

Studies have found that the reduced demethylation of histone H3K27 in the endothelial cells of CAD patients may enhance the expression of adhesion molecules, promoting inflammatory cell infiltration and serving as a potential epigenetic marker for CAD progression.

#### 5.4.6. Multi-Omics Integrated Diagnostic Tools

Integrating multi-dimensional epigenomic data, such as DNA methylation, miRNA expression, and histone modifications, can significantly improve the accuracy of CAD diagnosis. For example, a recently developed multi-omics scoring model successfully distinguished early CAD patients from healthy individuals, providing an efficient molecular diagnostic tool for clinical application [77].

Epigenomics demonstrates significant potential in CAD research. By comprehensively analyzing DNA methylation, histone modifications, and non-coding RNAs, researchers have uncovered molecular mechanisms underlying CAD and identified numerous epigenetic markers with diagnostic and prognostic value [78]. However, challenges such as sample size, data standardization, and detection technologies remain obstacles to clinical translation. In the future, with the integration of multi-omics technologies and the advancement of large-scale studies, epigenomics will play an increasingly vital role in the precise diagnosis and personalized treatment of CAD.

We compared the genomic technologies involved and their contribution to CAD research, as shown in Table 1.

## 6. Coronary Atherosclerosis-Related Gene Markers and Their Diagnostic Potential

### 6.1. Expression Changes in Gene Markers During Early CAD Stages

The expression changes in gene markers in the early stages of CAD provide critical clues for early disease detection. For example, mitochondrial and antioxidant enzyme paraoxonase, polymorphisms in genes that predominantly produce reactive oxygen species (NADH oxidase, endothelial-type nitric oxide synthase, and myeloperoxidase), i.e., genes for genetic markers of oxidative stress, have an important role in suggesting patients with an increased risk of CAD due to oxidative stress [79]. miRNAs play an important role in the regulation of pathophysiological processes such as cell adhesion, proliferation, lipid uptake, efflux, and inflammatory mediator production, which provides new molecular insights into their effects on these pathways in coronary atherosclerosis and helps to identify potential therapeutic approaches. The potential of miRNAs as biomarkers for diagnosis, prognosis, or therapeutic response in cardiovascular disease has been particularly enhanced by the realization [80] that miRNAs can be detected extracellularly, even in circulating blood [80]; also, *miR-33* controls vascular homeostasis and cardiac response to stress. In addition to *miR-33* and *miR-122*, single nucleotide polymorphisms near miRNA genes have been associated with abnormal lipid levels in human circulation. Some of these miRNAs, such as *miR-148a* and *miR-128-1*, target proteins involved in cellular cholesterol metabolism, such as the low-density lipoprotein receptor (LDLR) and ATP-binding cassette A1 (ABCA1) [80,81]; *miR-223*, which inhibits the *hmgs1*, *sc4mol*, and *srb1* genes, which are involved in HDL uptake and cholesterol production [82]; and nuclear factor (NF)-2, a single nucleotide polymorphism near the miRNA genes, have been linked to abnormal lipid levels in human circulation. Nuclear factor (NF)-κB signaling is also an important pathway for the activation of several other pro-inflammatory and pro-thrombotic factors. Two cytokine-responsive miRNAs (*miR-181b* and *miR-146a*) control NF-κB [83]; the most common miRNAs in CAD (*miR-1*, *miR-133a*, *miR-208a*, and *miR-499*) are prominently expressed in the heart and play important roles in cardiac physiology. Although a large number of miRNAs have been found to be expressed in ACS and stable CAD, *miR-1*, *miR-133*, *miR-208a*, and *miR-499* are usually considered as biomarkers of ACS [84], and these biomarkers, especially *miR-499*, whose concentration gradient levels correlate with myocardial injury, are the most probable diagnostics of ACS and stable CAD [85]. The abnormal expression of *JCAD* and *GUCY1A3* in vascular endothelial cells may reflect endothelial dysfunction, an early pathological event in atherosclerosis, even before clinical symptoms appear. Using highly sensitive molecular detection techniques, these changes can be identified at an early stage, enabling timely intervention.

### 6.2. Coupling with Specific Physiological Processes

#### 6.2.1. Regulation of Lipid Metabolism

Variations in *PCSK9* and *SORT1* affect core mechanisms of lipid metabolism, directly influencing the progression of arterial plaque formation. Circulating pcsk9 degrades the LDL receptor in lysosomes, leading to elevated ldl cholesterol. Camilla Gustafsen identified sortilin, encoded by the hypercholesterolemia risk gene *SORT1*, as a high-affinity sorting receptor for *PCSK9*. In healthy populations, circulating *PCSK9* is positively correlated with sortilin, suggesting that sortilin is involved in human *PCSK9* secretion. Sortilin was confirmed to be a key regulator of *PCSK9* activity [86].

By detecting the activity of these gene markers, individual risks of lipid metabolism abnormalities can be assessed, thereby optimizing treatment strategies.

#### 6.2.2. Amplification of Inflammatory Signals

Changes in the expression of inflammation-related genes, such as IL6R, reflect local inflammatory states, which are critical for assessing plaque stability and monitoring disease progression. Genetic analysis showed that the coronary artery disease-associated risk variant rs2832227 was associated with *BACH1* gene expression in patients’ carotid plaques, and endothelial Bach1 deficiency reduced turbulent blood flow or Western diet-induced atherosclerotic lesions in atherosclerotic mice, macrophage content in plaques, endothelial cell adhesion molecule (ICAM1 and VCAM1) expression, and decreased blood TNF-α due to IL-1β sub [87]. The NOS2A rs2297518 polymorphism was correlated with increased CCA-IMT, the IL1A rs1609682 polymorphism was correlated with susceptible plaques, and the HABP2 rs7923349 polymorphism showed a correlation. The GMDR analysis showed TNFSF4 rs1234313, IL1A rs1609682, TLR4 rs1927911, ITGA2 rs1991013, NOS2A rs2297518, IL6R rs4845625, ITGA2 rs4865756, and HABP2 rs7923349. There was significant gene–gene interactions between NOS2A rs8081248 and HABP2 rs932650 [88].

#### 6.2.3. Tools for Risk Stratification

Gene markers can also be used for risk stratification in CAD patients based on the association between their expression levels and disease severity. For instance, the following can be stated:

A high expression of *SORT1* may indicate a higher plaque burden. Sortilin, which is encoded by *SORT1*, plays an important role in the development of cardiovascular disease and has functions beyond regulating LDL cholesterol [58,89]. Gain-of-function mutations in *PCSK9* may be associated with high-risk patient populations. *PCSK9* potently reduced the level of *LDLR*. These markers provide a basis for risk prediction and therapeutic decision-making within the framework of precision medicine.

We compared key genetic markers of coronary atherosclerosis and their clinical roles, as shown in Table 2.

## 7. Applications of Gene Markers in Different Diagnostic Scenarios

### 7.1. Early Disease Detection

Gene marker detection can identify asymptomatic high-risk individuals, enabling early prevention. Early genetic association studies focused on polymorphisms situated within or near genes with a plausible biological role in the disease of interest (so-called candidate gene studies) [90].

In individuals carrying high-risk variants of *JCAD* or *GUCY1A3*, genetic screening combined with lifestyle interventions can effectively reduce the incidence of atherosclerosis. MiRNA’s potential as a diagnostic, prognostic, or therapy response biomarker for cardiovascular disease has been particularly increased by the realization that *miRNA* may be detected outside of cells, even in circulating blood [91]. A study showed that measuring *PCSK9* activity levels in blood samples could predict CAD risk 3–5 years in advance. *PCSK9* may be a potential target for pharmacologic treatment for this unmet medical need [86].

### 7.2. Risk Stratification

The expression patterns of gene markers can classify patients into different risk groups as follows:

(1) High-risk group: Patients with high *PCSK9* or *SORT1* activity may require more aggressive treatment strategies, such as a combination of *PCSK9* inhibitors and statins. Risk alleles in *SORT1*, *ABCG8, APOE,* and *LDLR* showed a statistically significantly higher frequency in blood relatives than in the 1000 Genomes Project [89].

(2) Intermediate-risk group: Patients with mild abnormalities in *JCAD* or *NOS3* expression could benefit from a combined strategy of lifestyle interventions and preventive medications. For this, there are preventive medications that can be taken; low-dose colchicine (0.5 mg/d orally) has been shown to safely lower major adverse cardiovascular events by 31% among those with stable atherosclerosis and by 23% after recent myocardial infarction [92].

(3) Low-risk group: Patients without significant abnormalities in genetic testing results may undergo long-term monitoring based on standard protocols. This dedicated pathway for a previously overlooked CAD population, with an accompanying registry, aims to improve outcomes through enhanced adherence to evidence-based secondary prevention and an additional diagnosis of modifiable risk factors observed [93].

### 7.3. Dynamic Monitoring and Treatment Guidance

The dynamic monitoring of gene markers can also guide treatment response evaluation.

Changes in *PCSK9* expression levels after statin therapy can reflect treatment efficacy and plaque stability. Coronary plaques that are prone to rupture and cause adverse cardiac events are characterized by large plaque burden, large lipid content, and thin fibrous caps. Statins can halt the progression of coronary atherosclerosis [94]. Statins are recommended as the first-line therapy for primary and secondary cardiovascular prevention in patients with hypercholesterinaemia and hypertriglyceridemia [95]. Reduced expression of inflammatory genes, such as *IL6R*, may indicate the effectiveness of anti-inflammatory therapies, such as IL-6 inhibitors. The expanding literature has led to understanding of the proatherogenic role for IL-6 in cardiovascular disease and thus the potential for IL-6 inhibition as a novel method for vascular protection [96].

## 8. Challenges and Future Directions

Gene markers associated with coronary atherosclerosis, such as *JCAD*, *GUCY1A3*, *PCSK9*, and *SORT1*, have demonstrated significant diagnostic and prognostic value in multiple studies. By analyzing the expression changes and functional characteristics of these genes, researchers have deepened their understanding of CAD pathogenesis and developed tools for early detection and risk stratification based on gene markers.

**Figure 3:**
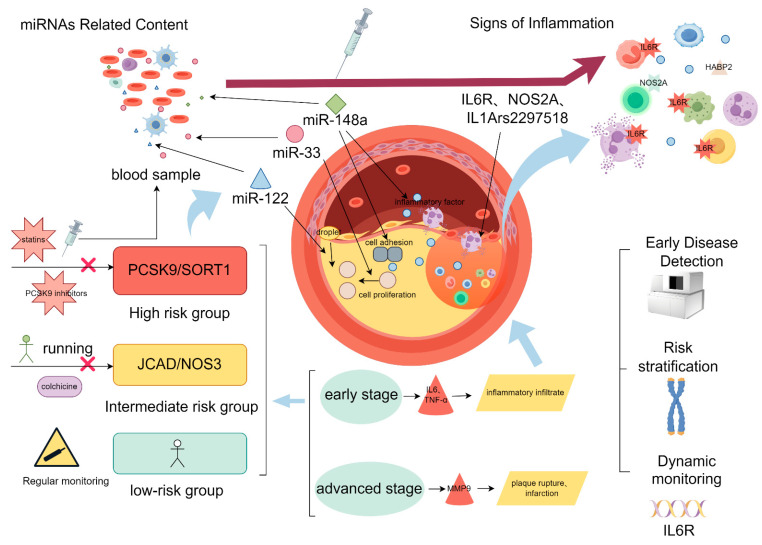
Clinical applications of genetic markers in coronary artery disease. This figure summarizes the integration of genetic markers and their clinical utility in diagnosing and managing coronary artery disease (CAD) across different stages. Genetic markers and miRNAs: Key markers such as *PCSK9*, *SORT1*, *JCAD*, *NOS3,* and associated miRNAs (*miR-148a*, *miR-33*, *miR-122*) are identified from blood samples. These markers are linked to lipid metabolism, vascular inflammation, and endothelial function, contributing to CAD risk stratification and management. Risk stratification: Patients are categorized into high-risk (e.g., *PCSK9/SORT1* mutations), intermediate-risk (e.g., *JCAD/NOS3* variants), and low-risk groups based on genetic profiles, guiding interventions such as statins, *PCSK9* inhibitors, or regular monitoring. Disease stage diagnosis: Genetic and inflammatory markers like *IL6R*, *NOS2A*, and *IL1Ars2297518* are utilized to differentiate between early-stage CAD (characterized by inflammatory infiltration with markers like IL-6 and TNF-α) and advanced-stage CAD (associated with plaque rupture and infarction, mediated by MMP-9). Clinical applications: These markers facilitate early disease detection, dynamic monitoring of disease progression, and personalized interventions to reduce CAD risk and improve patient outcomes.

However, the clinical translation of gene markers still faces challenges, including sample size, standardization of detection methods, and technological costs. In the future, with the integration of multi-omics data and the advancement of large-scale cohort studies, gene markers are expected to play a more significant role in the precise diagnosis and individualized treatment of CAD (Figure 3).

### 8.1. Challenges in the Application of Genomic Diagnosis in Coronary Atherosclerosis

#### Dynamic Changes in Gene Markers

(1) Changes During Disease Progression

Coronary atherosclerosis (CAD) is a multi-stage dynamically evolving pathological process involving early lipid deposition, chronic inflammatory responses, plaque stabilization, and later, plaque rupture. When the arterial endothelium encounters certain bacterial products or risk factors as diverse as dyslipidemia, vasoconstrictor hormones inculpated in hypertension, the products of glycoxidation associated with hyperglycemia, or pro-inflammatory cytokines derived from excess adipose tissue, these cells augment the expression of adhesion molecules that promote the sticking of blood leukocytes to the inner surface of the arterial wall [97,98]. As a major consequence of the inflammatory ferment underway in the early atheroma, SMCs migrate from the tunica media into the intima. These cells proliferate and elaborate a rich and complex extracellular matrix. Certain constituents of the extracellular matrix (notably proteoglycans) bind lipoproteins, prolong their residence in the intima, and render them more susceptible to oxidative modification and glycation (nonenzymatic conjugation with sugars). The expression and function of gene markers can vary significantly across different stages of the disease. For example, the following can be observed:

Early stages: Inflammation-related genes (e.g., IL6, TNF-α) show significantly elevated expression, reflecting endothelial dysfunction and inflammatory infiltration. Early atherosclerotic plaques, which consisted predominantly of SMCs, showed enhanced ECE expression in luminal endothelial cells and intimal SMC*s* [99].

Late stages: Genes associated with plaque rupture and thrombosis (e.g., MMP9) exhibit increased expression, while genes highly expressed in the early stages may return to baseline or decrease. In advanced atherosclerotic plaques, distinct ECE expression was found in accumulated macrophages and in endothelial cells of intraplaque microvessels, while luminal endothelial cells showed relatively weak immunoreactivity for ECE [99].

This dynamic nature necessitates the development of time-based marker monitoring models to capture key molecular events during CAD progression. However, most current studies focus on specific time points and lack longitudinal dynamic monitoring data, limiting the potential of genomic markers in precise CAD diagnosis.

(2) Changes Following Therapeutic Interventions

The impact of drug treatments and interventions on the expression of gene markers presents another significant challenge.

Statins: By inhibiting HMG-CoA reductase, statins lower LDL-C levels but may also affect the expression of *PCSK9* and other lipid metabolism-related genes, altering the detection outcomes of gene markers. Statins play a role in plaque regression with a reduction in lipid content. These drugs further stabilize atherosclerotic plaque with thickened fibrous caps and macrocalcification that serves to stabilize atheromas [100].

Anti-inflammatory therapies: Anti-inflammatory drugs, such as IL-6 inhibitors, may reduce the expression of inflammation-related genes, diminishing their utility in diagnosis and risk assessment during the later stages of treatment. Furthermore, Ma et al. found that Tan IIA (anti-inflammatory drugs) reduced vascular endothelial inflammation and prevented plaque formation via the COX-2/TNF-a/NF-κB signaling pathway. We have demonstrated for the first time that Tan IIA plays a vital role in attenuating atherosclerosis by downregulating COX-2 expression [100,101]. These results suggest that LLD, mostly statin in this study, might prevent the exaggeration of the G/G genotype-raising effect on inflammatory markers [102].

Currently, the long-term effects of drugs on the dynamic expression of gene markers, particularly the restoration patterns and clinical significance of gene expression after discontinuing therapy, remain insufficiently explored. This knowledge gap restricts the application of gene markers in personalized treatment and therapeutic monitoring.

### 8.2. Challenges at the Clinical Application Level

#### 8.2.1. Lack of Unified Clinical Standards and Diagnostic Guidelines

Genomic studies have identified numerous potential molecular markers for CAD diagnosis, but their clinical application is hindered by insufficient standardization.

#### 8.2.2. Differences in Detection Techniques

Significant variability exists among the gene detection platforms (e.g., Illumina, Ion Torrent) and data analysis pipelines used by different institutions, leading to inconsistent results. Currently used clinical risk algorithms, including the Framingham Risk Score, the Systematic Coronary Risk Evaluation 2 (SCORE2) system, and the Second Manifestations of Arterial disease 2 (SMART2), are based on traditional risk factors for cardiovascular disease and retrospectively predict future events with limited accuracy [103].

#### 8.2.3. Lack of Unified Thresholds

Diagnostic thresholds for gene markers are often determined independently by researchers, lacking unified clinical guidelines. This not only affects the comparability of results but also limits the broader implementation of gene markers in multi-center studies.

Organizations such as the International Organization for Standardization (ISO) are working to establish technical standards and guidelines for genomic diagnosis, but widespread adoption will require time and large-scale validation.

### 8.3. Challenges in Integrating Genomic Diagnosis with Traditional Methods

In clinical practice, genomic diagnosis needs to be integrated with traditional methods, such as imaging (e.g., coronary angiography, 2 MORE) and biochemical markers (e.g., LDL-C levels 2 MORE), to achieve higher diagnostic accuracy. However, this integration faces several challenges.

#### 8.3.1. Integration of Multidimensional Data

Several genetic loci have been reported to be significantly associated with CAD by GWASs. Nevertheless, the biological and functional effects of these genetic variants on CAD remain largely equivocal [80]. Genomic data often contain complex, multidimensional information, requiring advanced data analysis methods and algorithms for effective integration with imaging and biochemical data. Artificial intelligence and machine learning algorithms are considered potential tools to address this issue, but their clinical feasibility needs further validation.

#### 8.3.2. Optimization of Diagnostic Strategies

Large-scale clinical trials are needed to determine whether gene markers should be used as auxiliary indicators or core diagnostic criteria. Questions such as how to adjust treatment strategies based on changes in gene markers and how to quantify their weight relative to traditional indicators remain unresolved. Except for rare forms that follow a Mendelian inheritance pattern, CAD is a multifactorial trait caused by both genetic and environmental factors. Unlike single gene disorders, genetic studies of complex traits such as CAD are compounded by the lack of a perfect cosegregation between the risk allele and the phenotype and the high prevalence of the risk allele in the population. In a complex trait, the presence of a risk allele is neither necessary nor sufficient to cause the phenotype; hence, establishing causality is difficult [104].

Although genomic diagnosis holds tremendous potential for early detection and the risk assessment of coronary atherosclerosis, its application faces multiple challenges. The dynamic changes in gene markers and the impact of therapeutic interventions limit diagnostic reliability, while the lack of standardized guidelines and difficulties in integrating genomic methods with traditional approaches impede clinical adoption.

In the future, the establishment of dynamic monitoring models, the integration of multi-omics data, and the advancement of standardization efforts will be key to overcoming these challenges. These efforts will promote the comprehensive application of genomic diagnosis in CAD, laying the foundation for precision medicine.

## 9. Conclusions

Recent advances in CAD genetic research have identified promising markers like *JCAD/KIAA1462* and *GUCY1A3*, offering potential breakthroughs in early diagnosis and personalized medicine. However, clinical implementation faces significant challenges, including dynamic marker variability and the absence of standardized diagnostic guidelines, highlighting the need for comprehensive multicenter studies to establish unified protocols for widespread clinical adoption.

The future of CAD management lies in integrating multi-omics data and understanding cellular energy metabolism pathways, as demonstrated by *ATP5G1*’*s* role in mitochondrial function [105]. This gene’s influence on mitochondrial ATP synthesis and subsequent impact on cardiomyocyte energy supply illustrates the potential for targeted interventions based on individual genetic profiles. Research increasingly focuses on developing comprehensive models incorporating genomics, transcriptomics, proteomics, and metabolomics data while investigating critical gene–environment interactions such as dietary habits and air pollution exposure.

The development of dynamic risk assessment models integrating multiple data layers promises more precise early detection and monitoring capabilities. These advanced approaches enable real-time risk evaluation and personalized intervention strategies, potentially transforming CAD prevention and treatment through truly individualized medical care. Success in this endeavor requires continued collaboration across disciplines, including cardiovascular pathology, genetics, and bioinformatics, to translate these scientific advances into improved patient outcomes.

## Figures and Tables

**Table 1:** Genomic Techniques and Their Contributions to CAD Research.

Genomic Technique	Applications in CAD	Key Features/Strengths	Challenges
GWAS	Identification of CAD-associated genetic loci (e.g., *JCAD, SORT1*) across large populations	High-throughput; hypothesis-free approach	Small effect size of individual SNPs
Whole-Genome Sequencing (WGS)	Comprehensive genome-wide analysis	Detection of rare variants in non-coding regions	High cost and data complexity
Whole-Exome Sequencing (WES)	Focused on protein-coding regions	Identifies pathogenic mutations (e.g., *LDLR* in familial CAD); cost-effective	Misses regulatory and non-coding elements
Transcriptomics (RNA-seq)	Profiling of mRNA expression changes (e.g., *IL6R*)	Captures dynamic gene expression variability	Sensitive to sample preparation
Epigenomics	Identifies epigenetic regulatory mechanisms (e.g., *ANRIL*, miR-146a)	Uncovers complex regulatory mechanisms	Complex data interpretation
Liquid Biopsy	Early detection of CAD risk markers (e.g., *PCSK9*) from blood cfDNA or RNA	Minimally invasive	Expression variability due to interventions

**Table 2:** Key gene markers in coronary atherosclerosis and their clinical roles.

Gene Marker	Primary Function	Pathophysiological Role	Diagnostic and Therapeutic Potential	Relevant Studies
*JCAD/KIAA1462*	Endothelial adhesion and barrier function	Promotes endothelial dysfunction	Early detection of vascular endothelial damage	[26,28,43,44,45,46,47]
*GUCY1A3*	Vascular smooth muscle relaxation	Impairs smooth muscle relaxation	Risk prediction and potential target for vascular therapy	[29,48,49]
*PCSK9*	LDL-C metabolism	Elevates plasma LDL-C levels	Cholesterol-lowering therapies (e.g., *PCSK9* inhibitors)	[50,51,52,53,54]
*IL6R*	Inflammatory signaling	Drives chronic vascular inflammation	Biomarker for inflammation-driven CAD progression	[67]
*ANRIL*	Regulation of smooth muscle proliferation	Influences plaque stability	Risk stratification for cardiovascular events	[70,76]
*SORT1*	Lipid transport and metabolism	Regulates LDL metabolism	Risk stratification and therapeutic target for lipid disorders	[56,57,58]

## Data Availability

No new data were created or analyzed in this study. Data sharing is not applicable to this article.
